# Investigation of Youth Attitudes and Opinions Regarding HPV: A Systematic Review

**DOI:** 10.3390/healthcare14040439

**Published:** 2026-02-09

**Authors:** Eleftheria Papadopoulou, Elina Christiana Alimonaki, Anastasia Bothou, Anna Deltsidou, Maria Dagla, Christina Nanou, Giannoula Kyrkou

**Affiliations:** Department of Midwifery, University of West Attica, 12243 Athens, Greece; ria.1998@hotmail.com (E.P.); e.alimonaki@yahoo.com (E.C.A.); abothou@uniwa.gr (A.B.); adeltsidou@uniwa.gr (A.D.); mariadagla@uniwa.gr (M.D.); nanouxv@uniwa.gr (C.N.)

**Keywords:** HPV, vaccination, young people, youth, knowledge, attitudes, opinions, perceptions, HPV awareness

## Abstract

**Background:** Human papillomavirus (HPV) has been identified as the causative agent of cervical cancer and other cancers of the genital area, head and neck, on a worldwide scale. Despite the proven effectiveness of the vaccine in preventing HPV-related diseases, vaccination uptake remains low in many countries. It is crucial to understand how adolescents and young adults perceive HPV and its vaccine in order to design effective public health strategies and targeted awareness campaigns. **Aim:** This systematic review examined the attitudes, perceptions, and opinions of young people regarding HPV and HPV vaccination, with the goal of identifying factors that shape vaccine acceptance. **Methods:** A systematic literature search of PubMed and Scopus identified 293 articles published between January 2014 and February 2025. The review was conducted according to the Preferred Reporting Items for Systematic Reviews and Meta-Analyses (PRISMA 2020) guidelines. Eligible studies included primary qualitative and analytical cross-sectional research published in English that explored views on HPV and vaccination among individuals aged 15–24 years. Due to substantial heterogeneity in study design and outcome measures, findings were synthesized narratively and no meta-analysis was performed. The methodological quality and risk of bias of the included studies were assessed using the Joanna Briggs Institute (JBI) Critical Appraisal Checklists (2017) for qualitative and analytical cross-sectional designs. **Results:** Ten studies met the eligibility criteria, representing 15,776 young people across six countries. The included studies comprised eight cross-sectional surveys and two qualitative studies. Across these studies, knowledge and awareness of HPV and its vaccine were generally moderate, while vaccination coverage remained low. The highest awareness levels were reported in the United States (95.3% for HPV; 90.6% for the vaccine), whereas lower levels were noted in Brazil, Greece, and Italy. Influential factors associated with vaccine acceptance included parental attitudes, healthcare-provider recommendations, cultural norms, and the presence of misinformation. **Conclusions:** The level of awareness regarding HPV among adolescents and young adults in the included countries is moderate, yet the vaccination coverage remains low across the included countries. The provision of targeted educational interventions, in conjunction with the consistent guidance and training provided by healthcare professionals, is of considerable importance in order to increase vaccination rates. Findings should be interpreted in light of heterogeneity across studies and reliance on self-reported outcomes. Future research should adopt a mixed-methods approach to more effectively address the social, cultural and informational influences shaping young people’s perceptions, and to develop more effective communication strategies that promote HPV vaccination.

## 1. Introduction

Human papillomavirus (HPV) has been identified as one of the most prevalent sexually transmitted infections worldwide. Research studies in the field have identified approximately 200 subtypes of HPV, of which around 40 have been shown to lead to infection [1]. Young adults are particularly vulnerable to multiple strains associated with critical infections, while the risk of infection decreases with age and the development of immunity against the virus [2].

The clinical manifestations of HPV infection range from benign lesions caused by low-risk oncogenic HPV (LR-HPV) strains to severe forms of cancer, such as head and neck, oropharyngeal, penile, vulvovaginal, anal, and, most notably, cervical cancer caused by high-risk oncogenic HPV (HR-HPV) strains [3]. It is estimated that HR-HPV strains are the primary cause of approximately 5% of cancer cases [4].

However, HPV-related cancers can be prevented through vaccination, and the near-elimination of such cancers can be achieved through timely HPV vaccination for both genders. Increasing evidence suggests that the HPV vaccine is safe and provides a stronger immune response for maximum protection if administered between the ages of 9 and 14, before the onset of sexual activity [5].

Nevertheless, few countries have achieved satisfactory HPV vaccination coverage. Globally, according to the World Health Organization (WHO), the estimated vaccination coverage for HPV among women was 32%, with significant differences among individual programs across Europe, ranging from less than 5% to more than 90% in 2022 [6]. The observed disparity may be attributed to a number of factors, including a lack of information, preconceived ideas, social and cultural factors, and the propagation of inaccurate information through the mass media and the Internet [7].

Young people represent a social group that is constantly exposed to a wide range of information, especially through digital media and social networking platforms. This exposure often includes contradictory or incomplete messages about HPV infection and the HPV vaccine, which may influence how they think and feel about vaccination. Given that adolescence and young adulthood represent critical periods for the formation of health-related beliefs and behaviors, understanding how young people perceive HPV and its vaccine is essential for effective public health planning and intervention design.

According to a review by Iliadou et al. (2021), young people tend to have low to moderate awareness of HPV but are generally more positive toward vaccination. The authors emphasized the need for continued education of healthcare providers and highlighted the importance of identifying barriers that still prevent vaccine uptake [8]. However, existing reviews are often limited by a narrow geographical focus, the exclusion of qualitative evidence, or the inclusion of older studies that may not reflect recent changes in vaccination policies, communication environments, and digital information exposure.

Ramanadhan et al. (2020) [9], in a cross-sectional study, showed that many adolescents and their parents continue to have concerns about the HPV vaccine, largely due to insufficient information and limited access to reliable sources. The study emphasized that primary healthcare professionals and community-based organizations could play a larger role in raising awareness. Other qualitative studies have also found that some parents would consider vaccinating their children outside traditional clinical settings, which indicates a possible opportunity to increase coverage [10,11]. In several countries, school-based vaccination programs have already been implemented with encouraging results [12].

In Greece, available evidence shows that young adults (17–35 years) usually have a moderate knowledge regarding HPV transmission and prevention, with women appearing better informed than men. Reports indicate that just over half (52.3%) have been vaccinated, while the main reasons for refusing vaccination include inadequate information and fears related to side effects [13]. Despite relatively good general awareness of HPV, knowledge about transmission mechanisms and the preventive role of the vaccine remains limited [14].

Taken together, these findings highlight persistent gaps between awareness, knowledge, and actual vaccination behaviour among adolescents and young adults. Despite the availability of safe and effective vaccines, vaccination uptake remains suboptimal in many settings, underscoring the need for a comprehensive synthesis of contemporary evidence focused specifically on youth attitudes, perceptions, and decision-making processes.

### Aims and Objectives

The purpose of this systematic review was to synthesize and critically assess the existing research by examining the scope, methodological quality, and key findings of studies investigating young people’s attitudes and opinions toward HPV infection and vaccination. The review also aimed to identify the factors that shape young people’s knowledge, perceptions, and behaviours, including social, cultural, informational, and healthcare-related influences, with the broader goal of informing the development of evidence-based educational strategies and interventions that can support higher vaccination uptake and reduce HPV-related morbidity.

## 2. Materials and Methods

### 2.1. PICOS Eligibility Criteria

- (P) Population: Adolescents and young adults aged 15–24 years, from any country or cultural background, of any gender.
- (I) Intervention/Exposure: Attitudes, perceptions, opinions, and awareness related to human papillomavirus (HPV) infection and HPV vaccination, including exposure to information or educational messages, where reported.
- (C) Comparison: No predefined comparison group was required. Where applicable, descriptive comparisons within studies (e.g., between countries, genders, or vaccination status) were reported but not used as inclusion criteria.
- (O) Outcome: Reported attitudes, perceptions, opinions, awareness levels, and factors influencing HPV vaccination acceptance or hesitancy (e.g., parental influence, misinformation, cultural beliefs).
- (S) Study design: Primary qualitative studies and analytical cross-sectional studies.

### 2.2. Search Strategy

To investigate the above objective, a systematic review was conducted according to the methodology and guidelines of the Preferred Reporting Items for Systematic Reviews and Meta-Analyses (PRISMA statement) [15]. A structured bibliographic search was conducted in the PubMed and Scopus databases using the following search algorithm between January 2014 and February 2025. Our search strategy follows recommendations suggesting that a minimum of two electronic databases is adequate for comprehensive literature coverage as per Atkinson & Cipriani, 2018 [16].

The complete search strategy for Pubmed is outlined below as an example, with similar strategies adapted for Scopus ((“HPV”[Title/Abstract] OR “human papillomavirus”[Title/Abstract] OR “human papillomavirus vaccin*”[Title/Abstract] OR “hpv vaccin*”[Title/Abstract]) AND (“vaccine hesitancy”[Title/Abstract] OR “vaccine acceptance”[Title/Abstract] OR “attitude*”[Title/Abstract] OR “opinion*”[Title/Abstract] OR “HPV awareness”[Title/Abstract] OR “HPV knowledge”[Title/Abstract]) AND (“young adult*”[Title/Abstract] OR “young population*”[Title/Abstract] OR “youth”[Title/Abstract] OR “young people”[Title/Abstract] OR “aged 15–24”[Title/Abstract])) AND (2014/1/1:2025/2/25[pdat]).

The literature of the included studies was reviewed to identify and include studies that were not retrieved in the initial search. From the studies that emerged, the main elements were extracted, namely the type of study, the studied population, the purpose of the study, and the results regarding the opinions of young people.

The initially selected articles in this review were independently evaluated by two reviewers (EP and ECA). The results from the initial search were assessed based on the inclusion and exclusion criteria according to the title and abstract. After the duplicates were removed, the full text of the selected studies was evaluated by three reviewers (EP, ECA and AB). Discrepancies were resolved through discussion or consultation with a fourth author (GK) to reach a final decision.

### 2.3. Inclusion Criteria

Selection criteria were defined in advance following PRISMA recommendations. Studies were included if they met all of the following criteria:

1. Study design: observational, prospective, or retrospective studies, interventional studies, qualitative studies, or cohort-based research.
2. Population: Adolescents and young adults aged 15–24 years, following the World Health Organization (WHO, 2024) definition of “youth” [17].
3. Focus: primary empirical research examining attitudes, perceptions, opinions, or behavioral intentions related to HPV or HPV vaccination.
4. Publication period: January 2014 to February 2025, covering the period after the introduction of the 9-valent vaccine.
5. Accessibility: Available as full-text articles.
6. Language: English-language articles.

Although the predefined inclusion age range for this review was 15–24 years, some included studies involved broader age groups. In such cases, only data relevant to participants within or closely adjacent to the target age range were extracted and synthesized where possible. Specifically, studies including adolescents aged 14 years were retained when participants were attending secondary school and were developmentally comparable to the lower boundary of the inclusion criteria. The inclusion of participants aged 14 years may introduce differences related to consent processes, parental involvement, and autonomy in health decision-making, which can influence attitudes toward HPV vaccination. These factors were therefore considered when interpreting findings, particularly in relation to parental influence and vaccine decision dynamics, and caution was applied when comparing results across studies spanning different stages of adolescence.

In qualitative studies involving mixed participant groups (e.g., adolescents, young adults, and parents), only findings directly related to adolescents’ or young adults’ attitudes, perceptions, and vaccination decision-making were considered in the synthesis. Early adolescents aged 10–14 years were excluded from the review, as the focus was on attitudes, perceptions, and opinions regarding HPV and vaccination, which are more reliably captured among older adolescents and young adults who are able to articulate informed views and report vaccination-related decision-making experiences.

Despite the fact that interventional studies were eligible a priori according to the inclusion criteria, no interventional studies meeting all eligibility requirements were identified and included in the final synthesis.

### 2.4. Exclusion Criteria

Studies were excluded if they met any of the following criteria:

1. Non-primary research designs, including protocols, feasibility studies, reviews, systematic reviews, meta-analyses, editorials, case reports, case series, or modelling-only studies.
2. Population mismatch: Studies focusing exclusively on non-youth populations (e.g., parents, teachers, or healthcare professionals) or assessing proxy opinions about adolescents and young adults without their direct input.
3. Research focusing solely on prevalence or clinical outcomes without examining attitudes or perceptions;
4. Insufficient reporting of key methodological details.

### 2.5. Synthesis Methods

Due to substantial heterogeneity across included studies in terms of study design, populations, outcome measures, and data reporting, a quantitative meta-analysis was not appropriate. Therefore, findings were synthesized using a narrative synthesis approach, integrating quantitative results descriptively and summarizing qualitative findings thematically. This approach allowed comparison of patterns in attitudes, perceptions, awareness, and vaccination uptake across different contexts. Qualitative evidence was synthesized narratively by identifying and comparing recurring themes across studies, allowing convergence of findings related to knowledge, attitudes, and vaccination behaviors. For quantitative findings, awareness and vaccination uptake were categorized descriptively as low (<50%), moderate (50–74%), or high (≥75%) to support cross-study comparison, consistent with conventions commonly applied in public health research. These thresholds were used as heuristic, interpretive categories rather than validated cut-off points and were intended to facilitate comparative synthesis across studies with heterogeneous measurement tools.

For the purposes of this review, “awareness” was defined as having heard of HPV or the HPV vaccine, whereas “knowledge” referred to a deeper understanding of HPV transmission, health consequences, prevention, and vaccine-related information, as assessed by study-specific measures.

### 2.6. Protocol and Registration

The protocol for this systematic review was prospectively registered in the Open Science Framework (OSF) using the Generalized Systematic Review Registration template on 7 December 2025. The registration is currently under embargo in accordance with OSF policies.

## 3. Results

**Figure 1:**
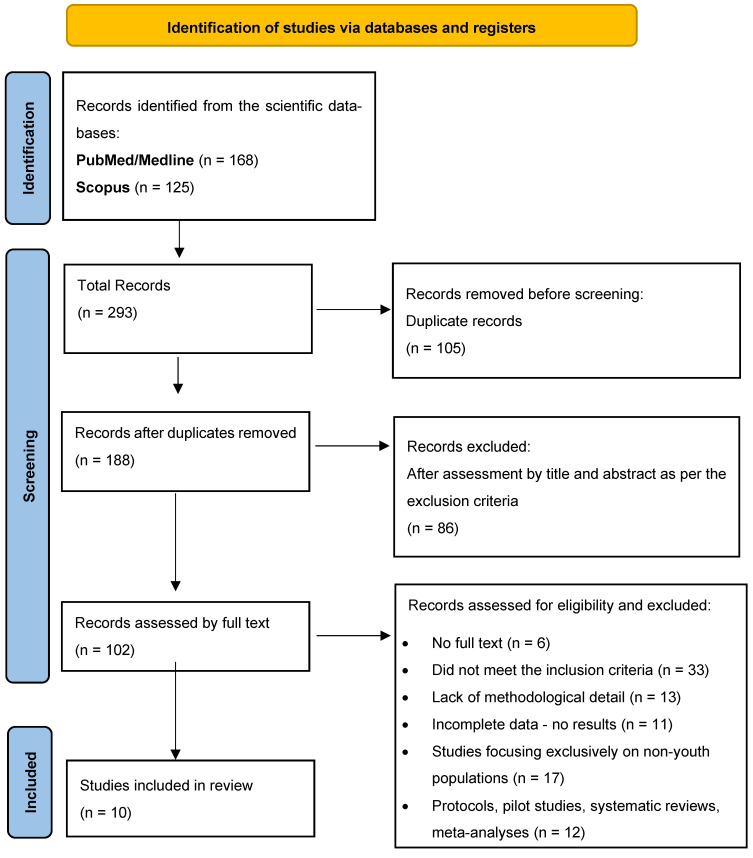
Flow diagram of articles included in the systematic review.

The database search across PubMed/Medline and Scopus initially identified 293 records. After removing duplicates, the remaining studies were screened by title and abstract according to the inclusion and exclusion criteria. Articles not meeting the eligibility criteria were excluded. The full texts of potentially relevant studies were reviewed in detail. Finally, ten studies met all inclusion criteria and were included in the synthesis. These consisted of both qualitative and cross-sectional quantitative studies, examining the attitudes, perceptions, and opinions of young people toward HPV infection and vaccination. The selection process is demonstrated in the PRISMA 2020 flow diagram (Figure 1).

### 3.1. Methodological Quality Assessment

Methodological quality was assessed independently by two reviewers using the appropriate Joanna Briggs Institute (JBI) Critical Appraisal Checklists (2017) according to study design. Any discrepancies were resolved through discussion, and when consensus could not be reached, a third reviewer was consulted. All studies ultimately included met acceptable methodological standards across key appraisal domains, including participant selection, measurement validity, and analytical rigor, as reflected in the individual appraisal results. The outcomes of the quality assessment are presented in Table 1 and Table 2.

The two qualitative studies, Glenn et al. (2021) [18] and Karamanidou & Dimopoulos (2018) [19] were evaluated using the JBI Critical Appraisal Checklist for Qualitative Research (10 items) [20], as shown in Table 1. Both studies showed an alignment between their research aims, methodological approach, and analysis. Glenn et al. fulfilled eight out of ten criteria and were considered at low risk of bias, with only minor gaps related to researcher reflexivity and positionality. Karamanidou & Dimopoulos met seven of the ten criteria and were assessed as having a moderate risk of bias, mainly due to limited reporting on researcher positioning and the absence of an explicitly stated ethics approval [18,19].

The remaining eight quantitative studies were assessed using the JBI Critical Appraisal Checklist for Analytical Cross-Sectional Studies (8 items) [21], presented in Table 2. Most studies satisfied the majority of the appraisal items, including clear inclusion criteria, appropriate measurement tools, and suitable methods. Six studies, Vaidakis et al. (2017) [22], Kasymova et al. (2019) [23], Kops et al. (2019) [24], Napolitano et al. (2016) [25], Mekonnen et al. (2024) [26], and Restivo et al. (2018) [27], were all judged to have a low risk of bias, with only minor limitations in reporting. Two studies, McCutcheon et al. (2017) [28] and Brunelli et al. (2025) [29], were classified as having a moderate risk of bias due to limited control for confounding and the absence of inferential statistical testing. Similarly, McCutcheon et al. (2017) were rated as moderate risk owing to their quasi-experimental design and restricted confounder control [28]. Although one study McCutcheon et al. (2017) [28] included a brief educational component, it was appraised as an analytical cross-sectional study because the intervention was not the primary focus, lacked a control group, and baseline descriptive outcomes formed the basis of synthesis.

Overall, methodological quality across all included studies was satisfactory, with most rated as low risk of bias. The diversity of contexts and populations enhances external validity, while minor methodological weaknesses do not compromise the reliability of the synthesized evidence. No study was excluded from synthesis based on quality.

While the majority of included studies were assessed as having a low risk of bias, a small number were classified as moderate risk. Findings derived from these studies were interpreted with appropriate caution in the synthesis and were considered primarily as supportive rather than definitive evidence, particularly where limitations related to confounding control or study design were present.

**Table 1:** Summary of quality appraisal of studies using the Joanna Briggs Institute (JBI) Critical Appraisal Checklist for Qualitative Research.

Author	Design	Q1. Is There Congruity Between the Stated Philosophical Perspective and the Research Methodology?	Q2. Is There Congruity Between the Research Methodology and the Research Question or Objectives?	Q3. Is There Congruity Between the Research Methodology and the Methods Used to Collect Data?	Q4. Is There Congruity Between the Research Methodology and the Representation and Analysis of Data?	Q5. Is There Congruity Between the Research Methodology and the Interpretation of Results?	Q6. Is There a Statement Locating the Researcher Culturally or Theoretically?	Q7. Is the Influence of the Researcher on the Research, and Vice- Versa, Addressed?	Q8. Are Participants, and Their Voices, Adequately Represented?	Q9. Is the Research Ethical According to Current Criteria or, for Recent Studies, and Is There Evidence of Ethical Approval by an Appropriate Body?	Q10. Do the Conclusions Drawn in the Research Report Flow from the Analysis, or Interpretation, of the Data?	Score	Overall Appraisal/Risk of Bias
Glenn et al. [18]	Qualitative	Y	Y	Y	Y	U	U	Y	Y	Y	Y	8/10	Include–Low RoB
Karamanidou & Dimopoulos [19]	Qualitative	Y	Y	Y	Y	U	U	Y	U	Y	Y	7/10	Include–Moderate RoB

Note. Y: yes (if it met quality criterion) indicated in green; U: unclear (if it did not mention relevant information) indicated in orange; RoB: Risk of Bias.

**Table 2:** Summary of quality appraisal of studies using the Joanna Briggs Institute (JBI) Critical Appraisal Checklist for analytical cross-sectional studies (last amended in 2017).

Author	Design	Q1. Were the Criteria for Inclusion in the Sample Clearly Defined?	Q2. Were the Study Subjects and the Setting Described in Detail?	Q3. Was the Exposure Measured in a Valid and Reliable Way?	Q4. Were Objective, Standard Criteria Used for Measurement of the Condition?	Q5. Were Confounding Factors Identified?	Q6. Were Strategies to Deal with Confounding Factors Stated?	Q7. Were the Outcomes Measured in a Valid and Reliable Way?	Q8. Was Appropriate Statistical Analysis Used?	Score	Overall Appraisal/Risk of Bias
Mekonnen et al. [26]	Cross-sectional	Y	Y	Y	Y	Y	Y	Y	Y	8/8	Include–Low RoB
Kasymova et al. [23]	Cross-sectional	Y	Y	Y	Y	Y	Y	Y	Y	8/8	Include–Low RoB
Kops et al. [24]	Cross-sectional	Y	Y	Y	Y	Y	Y	Y	Y	8/8	Include–Low RoB
Napolitano et al. [25]	Cross-sectional	Y	Y	Y	Y	Y	Y	Y	Y	8/8	Include–Low RoB
Restivo et al. [27]	Cross-sectional	Y	Y	Y	Y	Y	Y	Y	Y	8/8	Include–Low RoB
McCutcheon et al. [28]	Cross-sectional	Y	Y	Y	Y	U	N	Y	Y	6/8	Include–Moderate RoB
Brunelli et al. [29]	Cross-sectional	Y	Y	Y	Y	U	N	Y	Y	6/8	Include–Moderate RoB
Vaidakis et al. [22]	Cross-sectional	Y	Y	Y	Y	U	Y	Y	Y	7/8	Include–Low RoB

Note. Y: yes (if it met quality criterion) indicated in green; N: no (if it did not meet quality criterion) indicated in red; U: unclear (if it did not mention relevant information) indicated in orange; RoB: Risk of Bias.

### 3.2. Origin of Studies

The studies originated from six countries: two from Greece (Vaidakis et al., 2017; Karamanidou & Dimopoulos, 2018) [19,22], three from the United States (McCutcheon et al., 2017; Kasymova et al., 2019; Glenn et al., 2021) [18,23,28], two from Italy (Napolitano et al., 2016; Brunelli et al., 2025) [25,29], one from Brazil (Kops et al., 2019) [24], and one from Ethiopia (Mekonnen et al., 2024) [26].

This distribution provides representation from Europe, North and South America, and Africa, capturing varied sociocultural and healthcare contexts relevant to HPV vaccination. Table 3 summarizes the main characteristics of the ten studies included in this review.

### 3.3. Type of Study

Of the ten included studies, two employed qualitative methodologies (Glenn et al., 2021 [18]; Karamanidou & Dimopoulos, 2018 [19]), using interviews and focus group discussions to explore in depth young people’s beliefs, attitudes, and communication-related factors regarding HPV vaccination. In the study by Glenn et al. (2021), participants’ perspectives were elicited through a combination of semi-structured interviews with open-ended questions and focus group discussions, allowing both individual viewpoints and group interaction to inform the analysis [18]. Karamanidou & Dimopoulos (2018) similarly used focus groups guided by a structured discussion framework, supplemented by exposure to standardized informational stimuli (a documentary, an expert interview, and an informational leaflet), with alternating presentation order to enhance analytical rigor. In this study, limited quantitative questionnaire data were also collected to contextualize and support interpretation of the qualitative findings [19].

The remaining eight studies adopted quantitative analytical cross-sectional designs, primarily based on structured questionnaires administered in school, university, or community settings (Table 3). Notable heterogeneity was observed in study contexts, ranging from institution-based surveys to large-scale population-based datasets, such as the nationwide multicenter study conducted in Brazil by Kops et al. (2019) [24]. In the most recent Italian study by Brunelli et al. (2025), only data pertaining to participants aged 14–19 years (secondary school students) were extracted for synthesis, in order to maintain consistency with the predefined youth age range (15–24 years) of the present review; participants younger than 14 years were excluded from the analysis [29].

### 3.4. Study Purpose

All ten included studies examined adolescents’ and young adults’ knowledge, awareness, and attitudes toward human papillomavirus (HPV) infection and HPV vaccination. Beyond descriptive assessment, several studies also sought to identify factors influencing vaccine acceptance, refusal, or intention to vaccinate, including sociodemographic characteristics, gender differences, sources of information, and contextual influences (Table 3).

The qualitative studies were primarily designed to explore how beliefs, perceptions, social norms, and communication processes shape young people’s vaccination-related decision-making (Glenn et al., 2021 [18]; Karamanidou & Dimopoulos, 2018 [19]). These studies provided in-depth insight into informational needs, misconceptions, and trust-related barriers that are not easily captured through quantitative measures. In contrast, the quantitative cross-sectional surveys aimed to quantify levels of HPV-related knowledge and awareness, assess willingness to vaccinate and actual vaccination uptake, and examine associations with sociodemographic and behavioral variables across different national contexts (Kasymova et al., 2019 [23]; Kops et al., 2019 [24]; Napolitano et al., 2016 [25]; Vaidakis et al., 2017 [22]; Restivo et al., 2018 [27]; McCutcheon et al., 2017 [28]; Brunelli et al., 2025 [29]; Mekonnen et al., 2024 [26]).

Across all studies, the overarching purpose was not only to describe the current state of HPV-related knowledge and attitudes among adolescents and young adults, but also to identify key determinants that may facilitate or hinder vaccine acceptance. Collectively, these aims support a more comprehensive understanding of why gaps between awareness and vaccination uptake persist and highlight areas where targeted public health and communication strategies may be most effective.

### 3.5. Study Sample

Overall, the eligible studies included a sample of 15,776 young people. Sample sizes ranged from small qualitative cohorts to large national surveys. The largest samples were reported in the nationwide Brazilian study by Kops et al. (2019) [24] and the school-based survey conducted in Greece by Vaidakis et al. (2017) [22], whereas the qualitative studies included smaller, purposively selected participant groups (Glenn et al., 2021 [18]; Karamanidou & Dimopoulos, 2018 [19]).

The age range of participants predominantly covered adolescence and young adulthood, with some studies focusing exclusively on female populations (Mekonnen et al., 2024 [26]; Restivo et al., 2018 [27]) or male populations (Napolitano et al., 2016 [25]; McCutcheon et al., 2017 [28]). This variation in sample composition enabled the identification of gender-specific patterns in HPV-related knowledge, attitudes, and vaccination uptake. Table 4 shows the demographic characteristics of the study samples.

### 3.6. Study Findings

Findings were synthesized narratively by identifying recurring patterns in awareness, attitudes, and vaccination uptake across countries and study designs.

#### 3.6.1. Knowledge and Awareness of HPV and HPV Vaccination

The results of the included studies indicate that adolescents and young adults generally demonstrate moderate levels of surface awareness of HPV infection and vaccination (having heard of HPV or the vaccine), whereas functional knowledge (including understanding of HPV transmission, cancer risk, and prevention) remains limited, and vaccination coverage remains consistently low across most countries and study populations, with variation linked to geographic and sociodemographic factors.

**Figure 2:**
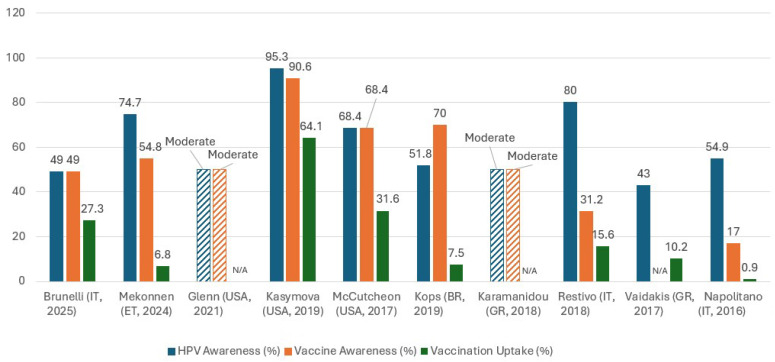
Overview of HPV awareness, vaccine awareness, and vaccination uptake among adolescents and young adults across the included studies. Quantitative studies are presented using reported percentages. Qualitative studies [18,19] are displayed using descriptive categorical indicators (e.g., low or moderate) derived from thematic findings, rather than numerical values. Missing bars (N/A: Not Available) indicate metrics not reported in the source study [18,19,22,23,24,25,26,27,28,29].

An illustration of these variations is provided by Figure 2, which presents a comparative overview of HPV awareness, vaccine awareness, and vaccination uptake reported across the ten included studies. In the majority of quantitative studies, awareness (defined as having heard of HPV or the HPV vaccine) was found to be moderate. However, this did not translate into adequate conceptual understanding, as substantial misconceptions regarding HPV-related cancer risk, male susceptibility, and vaccine effectiveness were frequently reported. Moreover, vaccination uptake remained low across nearly all populations examined. To support comparability across quantitative studies, awareness and uptake were categorized as low (<50%), moderate (50–74%), or high (≥75%), in line with interpretive conventions frequently adopted in public health and behavioral science research (WHO Europe, 2021; ECDC, 2021) [30,31]. This categorization enabled more consistent interpretation of findings derived from studies with different designs and measurement tools. For the two qualitative studies (Glenn et al., 2021; Karamanidou & Dimopoulos, 2018), awareness levels are represented descriptively using categorical indicators (e.g., low or moderate), based on the authors’ thematic interpretations of participants’ narratives [18,19]. These qualitative indicators are included for illustrative purposes only and do not represent numerical measurements or empirically derived estimates. This approach allows qualitative and quantitative evidence to be presented alongside each other in Figure 2, while preserving their distinct methodological characteristics and avoiding over-quantification of narrative findings.

Of the quantitative studies, Kasymova et al. (2019) in the United States recorded the highest levels of awareness and vaccination, with 95.3% of students being aware of HPV and 90.6% aware of the vaccine [23]. In contrast, Kops et al. (2019) in Brazil reported a mean knowledge score of 51.8%, indicating moderate accuracy of HPV-related knowledge, accompanied by a very low vaccination rate of 7.5% [24]. In Greece, Vaidakis et al. (2017) found that, while 42.8% of adolescents had heard of HPV, more than 60% did not recognise its association with cervical cancer, highlighting limited disease-specific knowledge despite moderate awareness [22].

In Italy, Napolitano et al. (2016) reported that 54.9% of young men were aware of HPV, yet only 0.9% had received the vaccine [25]. Similarly, Brunelli et al. (2025) found that 49% of Italian adolescents aged 14–19 years were aware of the HPV vaccine, while 27.3% had received at least one dose [29]. In Ethiopia, Mekonnen et al. (2024) reported that HPV infection had been heard of by 72.7% of university students and the vaccine was known about by 52.5% of them, yet vaccination uptake remained low at 6.8%, indicating a substantial gap between awareness and vaccination behaviour [26]. Because vaccination status was self-reported in most quantitative studies, vaccination uptake estimates should be interpreted cautiously, as recall and social-desirability bias may influence reported coverage.

#### 3.6.2. Attitudes, Beliefs, and Willingness to Vaccinate

In the qualitative study by Glenn et al. (2021), many young adults expressed uncertainty regarding the benefits of the HPV vaccine and concerns about potential side effects, particularly in relation to male vaccination. These findings reflected limitations in knowledge rather than a lack of awareness. Parental influence and recommendations from healthcare providers were reported as strong determinants shaping vaccination-related decisions [18].

Similarly, Karamanidou & Dimopoulos (2018) found that Greek adolescents, young women, and mothers exhibited low to moderate awareness, alongside an average knowledge score of 5.4 out of 10. Participants frequently expressed uncertainty and misconceptions regarding vaccine safety and effectiveness, indicating ambivalence and mistrust that influenced attitudes toward vaccination [19].

Among male student-athletes in the United States, McCutcheon et al. (2017) reported that 68.4% were aware of HPV, yet the same proportion remained unvaccinated. Following a brief educational exposure, improvements were observed in HPV-related knowledge and willingness to vaccinate, underscoring the role of targeted information in shaping attitudes rather than awareness alone [28].

#### 3.6.3. Factors Influencing Knowledge, Attitudes, and Vaccination Behaviors

Across studies, vaccination coverage remained consistently low despite moderate awareness levels, suggesting that awareness alone was insufficient to drive vaccine uptake. In Italy, Restivo et al. (2018) reported that only 15.6% of young women had received at least one dose of the HPV vaccine, while 84.4% remained unvaccinated [27]. Interestingly, this study identified an inverse association between educational attainment and vaccination outcomes, with participants of lower educational levels demonstrating higher awareness and higher initiation and completion rates.

In Greece, the low vaccination uptake (10.2% of girls) reported by Vaidakis et al. (2017) occurred despite moderate awareness levels, pointing to additional barriers such as limited access, cultural reservations, or insufficient healthcare engagement [22]. Similarly, in Brazil, Kops et al. (2019) observed very low vaccination coverage in a national sample, despite moderate knowledge scores, highlighting the potential role of systemic and structural barriers [24].

Across settings, female participants and individuals exposed to healthcare or educational information tended to demonstrate greater awareness of and acceptance of the HPV vaccine. Commonly reported barriers to vaccination included misinformation, safety concerns, and low perceived personal risk [26,29]. These findings indicate that structural factors, communication quality, and healthcare accessibility interact with individual knowledge and attitudes to shape vaccination behaviours among adolescents and young adults.

## 4. Discussion

HPV vaccination remains a fundamental public-health strategy. It is used to prevent HPV-related diseases. This is particularly important for preventing cervical cancer [32]. The effectiveness of such programs depends on more than just vaccine availability. It also depends on public awareness, attitudes, and uptake [33,34,35]. This systematic review examined the perspectives of adolescents and young adults aged 15–24 years in six countries to explore their understanding of HPV infection and HPV vaccination. Of the 293 articles that were identified, the inclusion criteria were met by ten, with 15,776 participants being represented. The findings overall suggest that, despite the broad acknowledgement of the public health importance of HPV vaccination, there are moderate levels of surface awareness, while comprehensive, functional knowledge remains limited. This indicates that awareness alone does not constitute a sufficient baseline for informed vaccination decision-making. Furthermore, vaccination coverage remains consistently low. Considerable variation was noted between countries. There was also variation between different methodological approaches.

Building on these findings, the present review offers several contributions beyond existing syntheses. Unlike earlier reviews that primarily focused on vaccination coverage or general knowledge, this review integrates both quantitative and qualitative evidence to capture not only levels of awareness and uptake but also the social, communicative, and contextual mechanisms shaping vaccination decisions among adolescents and young adults. In addition, the extended time frame (2014–2025) reflects changes in HPV vaccination recommendations, including the increasing inclusion of males, as well as the growing influence of digital information environments. By incorporating evidence from diverse settings, including Southern Europe, Latin America, and sub-Saharan Africa, this review provides a broader cross-contextual perspective on youth attitudes toward HPV vaccination than has been previously reported. Nevertheless, it should be acknowledged that several world regions, including South Asia, East Asia, and the Middle East, were not represented in the included studies. As a result, the findings should be interpreted with caution, and broad global generalizations should be avoided until further evidence from these regions becomes available.

### 4.1. Differences in Knowledge and Awareness

Across studies, awareness of HPV was consistently higher than comprehensive knowledge, indicating that having heard of HPV or the vaccine often coexisted with substantial misconceptions, including limited understanding of HPV-cancer links, male susceptibility, and vaccine effectiveness. This gap may partially explain the persistently low vaccination uptake observed across settings.

Levels of HPV knowledge and vaccine awareness differed widely across settings. Overall, higher awareness and more accurate HPV-related knowledge tended to be reported in contexts where adolescents and young adults have greater exposure to structured health education initiatives and more consistent access to preventive health information, particularly within school and university environments [23,36]. Similar patterns are observed in studies from the UK and Sweden, where adolescents’ and young adults’ awareness frequently exceeds 80% [37].

In contrast, studies conducted in settings where health education is less systematically delivered, or where access to preventive healthcare and reliable information is more uneven, reported lower knowledge accuracy and weaker understanding of HPV-related risks. Comparable patterns have been described in several low- and middle-income contexts, where limited access to preventive healthcare services and educational resources can constrain HPV knowledge and awareness [24,38,39]. In India, it was found by Shetty et al. [40] that HPV and its association with cervical cancer were recognised by only 59.7% of women.

Moderate to low levels of knowledge have also been reported in Mediterranean countries, reflecting persistent gaps in understanding key HPV-related outcomes, such as the association between HPV infection and cervical cancer (Napolitano et al., 2016 [25]; Vaidakis et al., 2017 [22]). These findings are consistent with broader evidence from Southern Europe emphasizing the continued need for enhanced targeted education and structured prevention messaging for adolescents and young adults [37].

Recent evidence supports these observations and further highlights subgroup differences. In Italy, awareness- and prevention-related understanding appeared more developed among older adolescents and females compared with their peers (Brunelli et al., 2025 [29]). In Ethiopia, despite relatively broad awareness of HPV infection, vaccine uptake remained limited, suggesting that knowledge and attitudes alone may not be sufficient to translate into vaccination uptake in the absence of supportive delivery systems and trusted health communication, as discussed within the broader public health literature (Mekonnen et al., 2024 [26]). Notably, higher knowledge and more favorable attitudes were more commonly reported among health-science students and those receiving information from healthcare professionals, reinforcing the role of trusted sources in shaping prevention beliefs and vaccine-related understanding [25].

### 4.2. Vaccination Uptake and Determinants

Vaccination uptake showed considerable variation across countries. In general, uptake appeared more favorable in contexts characterized by more established national immunization infrastructure, preventive health services, and youth-facing communication strategies, whereas lower uptake was reported in settings where vaccine delivery systems are less integrated or where access is constrained by practical, structural, or socioeconomic barriers (Kasymova et al., 2019 [23]; Kops et al., 2019 [24]; Restivo et al., 2018 [27]; Vaidakis et al., 2017 [22]). These patterns suggest that vaccination behavior is shaped not only by individual awareness and attitudes but also by broader system-level determinants, including accessibility, affordability, and consistent healthcare engagement [41]. In addition, because vaccination coverage was primarily based on self-report across included studies, cross-country differences in uptake may partly reflect measurement bias rather than true differences in coverage, and should therefore be interpreted with caution.

Differences in HPV knowledge, attitudes, and vaccination behaviors across studies may partially reflect differences in vaccine accessibility and delivery models across countries, as suggested by the contextual characteristics reported in the included studies. In settings where HPV vaccination is integrated into national immunization programs, offered free of charge, or delivered through school-based initiatives, such as in the United States, higher levels of awareness and vaccination uptake were observed (Kasymova et al., 2019 [23]). In contrast, studies conducted in contexts with more fragmented delivery systems or age-restricted reimbursement policies, including parts of Southern Europe and Latin America, reported lower vaccination uptake despite moderate awareness (Vaidakis et al., 2017 [22]; Restivo et al., 2018 [27]; Kops et al., 2019 [24]).

In low-resource settings, such as Ethiopia, limited vaccine availability and emerging immunization infrastructure may further constrain uptake, even when awareness is relatively high (Mekonnen et al., 2024 [26]). Taken together, these findings suggest that structural accessibility interacts with individual knowledge and attitudes, highlighting that favorable knowledge and positive attitudes alone may be insufficient to achieve high vaccination coverage without supportive health system policies. Beyond structural accessibility, interpersonal communication and trusted information sources also play a central role in shaping vaccination decisions.

Importantly, these findings suggest that HPV vaccination uptake among adolescents and young adults should not be viewed mainly as a matter of individual choice or insufficient knowledge. Instead, uptake appears to be strongly influenced by how vaccination is organized and supported at the health-system and policy level. Factors such as whether the vaccine is publicly funded, how it is reimbursed, its inclusion in national immunization schedules, and the availability of school-based delivery programs play a crucial role in enabling equitable access. In this respect, the cross-country differences observed in this review are consistent with broader global public health priorities, including the World Health Organization’s strategy for cervical cancer elimination, which identifies high HPV vaccination coverage achieved through organized, publicly supported immunization programs as a central component of prevention.

The qualitative findings of Glenn et al. (2021) [18] demonstrated that parental views and the recommendations made by healthcare providers played a pivotal role in the decision-making process regarding vaccines among U.S. students. Communication gaps in Greece were identified by Karamanidou & Dimopoulos (2018) [19], where uncertainty, mistrust of media messages and mixed information were found to be constraining factors in vaccine acceptance. These findings are consistent with a growing body of evidence underscoring the pivotal role of reliable communication and trusted healthcare professionals in promoting vaccination [42,43].

Across settings, the most common reasons for vaccine hesitancy remain misinformation, safety concerns and low perceived personal risk [26,44]. Although formal behavioral frameworks such as the Health Belief Model or the Theory of Planned Behavior were not the primary focus of this review, several of the identified findings can be conceptually aligned with key constructs from these models. Across studies, perceived susceptibility, safety concerns, social influence from parents and healthcare providers, and access-related barriers emerged as important factors associated with vaccination decisions. These observations suggest that established behavioral theories may offer a useful interpretive lens for understanding youth attitudes toward HPV vaccination, even though the included studies did not consistently apply such frameworks.

When interpreting differences across studies, it is also important to consider the broader cultural, healthcare-system, and policy contexts in which the research was conducted. Cultural attitudes toward sexual health, gender norms, and preventive care can influence how HPV infection and vaccination are understood, particularly in settings where discussions around sexually transmitted infections remain sensitive. In addition, healthcare-system factors such as access to primary care, the involvement of healthcare professionals in preventive counselling, and the availability of school-based vaccination programs differ considerably between countries.

Vaccination policies, including whether the HPV vaccine is publicly funded, offered free of charge, or subject to age restrictions, have been shown in previous research to influence uptake and may help contextualize the variation observed across the included studies. Consequently, variations observed across studies are likely shaped not only by individual knowledge and attitudes, but also by structural and contextual conditions. Acknowledging these differences is essential for interpreting cross-country findings and for designing interventions that are appropriately tailored to local healthcare and cultural environments.

From a practical perspective, these findings reinforce the need to prioritize policy-level and system-based interventions alongside individual-focused education. In this context, effective strategies are likely to be context-specific and multifaceted, combining school-based vaccination programs, targeted educational initiatives for young men, and active engagement of primary care providers in preventive counselling. In parallel, the strategic use of digital and social media platforms may help counter misinformation and deliver culturally appropriate messages tailored to adolescent and young adult populations. These findings align with established behavioral frameworks, such as the Health Belief Model, in which perceived susceptibility, perceived benefits and barriers, and cues to action (healthcare-provider recommendations) are central determinants of vaccination behavior.

### 4.3. Implications for Educational Strategies and Interventions

Overall, the findings of this review suggest that increasing knowledge and awareness of HPV and its vaccine, while important, is not sufficient on its own to achieve higher vaccination uptake among adolescents and young adults. Although most studies reported moderate levels of awareness, vaccination decisions were often shaped by concerns about vaccine safety, uncertainty regarding benefits, and low perceived personal risk. These observations indicate that effective educational strategies should not focus solely on information delivery, but also address the attitudes, beliefs, and contextual factors that influence behavior. Approaches that involve healthcare professionals, engage parents, and provide clear and consistent messages tailored to young people appear particularly relevant, given their influence across multiple studies. Educational interventions delivered through schools, digital platforms, or community settings may be especially valuable when combined with accessible vaccination services, helping to translate awareness into actual vaccine uptake.

### 4.4. Methodological Considerations

The studies included in the review used a variety of methodological approaches. The majority of quantitative studies, including those undertaken by Kasymova, Vaidakis, Kops, Napolitano, Restivo, Mekonnen, and Brunelli, relied on structured and validated questionnaires [22,23,24,25,26,27,29]. McCutcheon et al. (2017) [28] used a pre–post educational format to descriptively explore changes in HPV-related knowledge and vaccination intentions among male college athletes following a brief informational session. The qualitative studies (Glenn, Karamanidou & Dimopoulos) used semi-structured interviews and focus groups, which provided more detailed information about people’s beliefs, worries, and the communication processes that influence attitudes to vaccination [18,19]. It should be noted that a proportion of the evidence on which these interpretations are based originates from studies that have been evaluated as having a moderate risk of bias, particularly those utilizing descriptive or quasi-experimental designs. Consequently, these findings should be interpreted with appropriate caution and considered alongside the more robust evidence derived from low-risk studies.

In combination, these studies illustrate the significance of integrating qualitative and quantitative evidence to enhance our comprehension of not only knowledge levels but also the extensive cultural and social influences that impact vaccination behaviours [45].

### 4.5. Implications

Overall, this review discloses that, despite an improvement in awareness of HPV vaccination over time, considerable gaps persist in the translation of knowledge into action. Across the included countries, vaccine uptake continues to be influenced by cultural norms, misinformation, and healthcare access. It has been highlighted by the two most recent studies (Brunelli, 2025; Mekonnen, 2024) that uptake remains limited even among well-informed youth populations, suggesting that vaccine compliance cannot be ensured by knowledge alone [26,29].

The consistent gap identified between HPV awareness or knowledge and actual vaccination uptake underscores the need for interventions that help translate information into action. Although many young people are aware of HPV and its vaccine, this awareness does not always lead to vaccination, indicating that educational efforts alone may be insufficient. Strategies that combine education with practical delivery mechanisms may therefore be more effective. School-based vaccination programs could reduce missed opportunities by reaching adolescents at an earlier stage, while digital health education may offer flexible and accessible ways to provide accurate, age-appropriate information. In addition, communication delivered by healthcare professionals appears to play a key role in addressing safety concerns and reinforcing trust in vaccination. Taken together, these approaches highlight the importance of integrating educational initiatives with healthcare-system support in order to improve HPV vaccination uptake.

### 4.6. Strengths and Limitations

This systematic review has several strengths. A broad synthesis of literature examining attitudes, awareness, and vaccination behaviours of adolescents and young adults across six countries and four continents is provided. It incorporates evidence from both qualitative and quantitative studies. This offers a well-rounded perspective on HPV-related knowledge and it also looks at the social and cultural influences shaping vaccination decisions. The quality of the studies was assessed using the Joanna Briggs Institute (JBI) Critical Appraisal Checklists, with a systematic evaluation of methodological rigour and risk of bias being ensured.

However, it is important to recognize the limitations of this review. Considerable heterogeneity in study designs, measurement tools, outcome definitions, and population characteristics precluded the conduct of a meta-analysis. In particular, the instruments used to assess knowledge, awareness, attitudes, and vaccination status varied substantially across studies, limiting the direct comparability of findings and the synthesis of results across settings. In addition, most included studies relied on self-reported measures of awareness, attitudes, and vaccination status, which may be subject to recall bias or social-desirability bias. As a result, reported levels of knowledge and vaccination uptake may be overestimated in some settings, and findings should therefore be interpreted with caution. Moreover, the majority of the included studies were cross-sectional, which restricts the possibility of causal inference. This review did not systematically apply a specific behavioral framework, as this was not a predefined objective; however, future reviews may benefit from explicitly integrating theoretical models to further contextualize determinants of HPV vaccination attitudes. Finally, only English language publications from January 2014 to February 2025 were included, meaning that relevant evidence published in other languages or from other regions may have been missed.

## 5. Conclusions

This systematic review demonstrates that, although moderate awareness of HPV infection and vaccination is generally exhibited, detailed knowledge remains limited among adolescents and young adults, and consistent low vaccination coverage is still observed across countries. Higher awareness and uptake were observed in some contexts. These include the United States. However, substantial informational and implementation gaps persist in Southern Europe, Latin America, and sub-Saharan Africa. It has been found by recent studies that even when young people are aware of HPV and its vaccine, vaccination compliance is not ensured by knowledge alone, highlighting the influence of sociocultural, educational, and healthcare-related factors on vaccine acceptance. Public health strategies must prioritize targeted education, establish reliable communication channels, improve access to preventive healthcare services in a timely manner, and implement initiatives aimed at strengthening vaccine confidence among adolescents and young adults.

Future research should expand the geographical scope of evidence and apply mixed-method designs combining quantitative and qualitative approaches. This would allow for a deeper understanding of young people’s perceptions and the social, psychological, and cultural factors shaping their attitudes toward HPV vaccination, ultimately supporting more effective global prevention strategies.

Key public health implications include:

(1) The need for adolescent and youth-centered educational interventions, particularly in settings with low vaccination uptake.
(2) The importance of healthcare provider engagement and school-based or community-based programs to translate awareness into vaccination action.
(3) The value of tailored communication strategies, including digital and social media approaches, to counter misinformation and promote informed decision-making.

## Figures and Tables

**Table 3:** Eligible studies’ characteristics.

First Author, Year, Country	Study Type	Data Collection Method	Population, Age (Years)	Results	Conclusions
Brunelli et al., 2025, Italy [29]	Cross-sectional observational study	Structured, self-administered questionnaire distributed in secondary schools; based on validated items and pilot-tested. Part of the national project ESPRIT from the Italian National Center for Disease Control and Prevention	Adolescents aged 14–19 years, both sexes; recruited from Italian secondary schools	The level of awareness about HPV was found to be 66%. 37% recognized the link to cervical cancer; Vaccine awareness was 49.0% and 27% reported being vaccinated. Knowledge and vaccine awareness were significantly higher among females. 27.3% of cases had received at least one dose of vaccine. Attitudes were generally positive, though misconceptions and information gaps remained prominent.	The study revealed suboptimal knowledge and awareness about HPV and its vaccine among Italian adolescents, despite positive attitudes toward prevention. The authors recommend enhanced school-based education and targeted awareness campaigns to improve understanding and vaccination uptake.
Mekonnen et al. 2024, Ethiopia [26]	Cross-sectional institutional study	Pre-tested structured self-administered questionnaire adapted from previous validated tools; reliability confirmed	Female university students aged 19–23	Knowledge about HPV was 72.7% and 52.5% were aware of the HPV vaccine; 57% expressed positive attitudes toward HPV vaccination, and 43.5% believed the HPV vaccine was safe and effective. Vaccination uptake was 6.8% (43 out of 633). All participants had never been tested for HPV.	The study concluded that knowledge, attitudes were at a good level, but vaccination coverage remains low among Ethiopian university students, highlighting the need for targeted educational interventions, health-provider engagement, and integration of HPV education into universities to improve vaccine acceptance.
Glenn et al. (2021), USA [18]	Qualitative study (semi-structured interviews/focus groups)	Semi-structured interviews and focus groups with college students and student-health providers; mostly in-person, some by phone, audio-recorded, transcribed, and analyzed thematically in ATLAS.ti	College students aged 18–24	Young adults’ vaccination decisions were influenced by parents and healthcare providers. Many lacked knowledge about male vaccination benefits and expressed concerns about vaccine safety and side effects. Barriers included uncertainty about vaccination status, insurance coverage, and scheduling conflicts. Providers’ recommendations were influenced by system-level factors such as clinic infrastructure and visit priorities.	Provider recommendations and addressing logistical barriers are essential to improve vaccine uptake among college students. The success of multilevel interventions in improving vaccination rates among young adults eligible for catch-up immunization is imperative.
Kasymova et al. (2019), USA [23]	Cross-sectional study (HPV-K18 questionnaire)	Paper-and-pencil self-administered classroom survey among undergraduate students; anonymous participation; data collected in classroom settings.	College students, mean age 19.7 (80.9% female)	95.3% knew about HPV and 90.6% about vaccination. Ethnicity did not affect knowledge, but gender differences were significant for females (97% vs. 88%, *p* = 0.005). 64.1% had received at least one dose. Cognitive gaps regarding HPV in men have been identified. Confusion was found about HPV and genital herpes. Only 13% of people were aware that HPV does not cause genital herpes. Female and white participants, as well as those vaccinated, demonstrated higher knowledge.	University-level health programs should reinforce HPV education, especially for male and minority students.
Kops et al. (2019), Brazil [24]	National multicenter cross-sectional study (POP-Brazil Study)	Face-to-face standardized interviews conducted by trained healthcare professionals from 119 public primary care units	Young adults aged 16–25 (74% female)	Mean correct answer rate was 51.79%. Women had significantly higher knowledge scores than men (*p* = 0.0003). Approximately 46.30% believed that HPV has signs and symptoms. Only 7.48% were vaccinated against HPV (95% CI 6.69–8.27).	The combination of mass media and counseling by health professionals increases awareness and vaccination coverage. Communication strategies should also consider the involvement of men.
Karamanidou & Dimopoulos (2018), Greece [19]	Qualitative study (semi-structured interviews/focus groups)	Qualitative focus groups with unvaccinated adolescent girls, young women, and mothers; pre-group questionnaires collected sociodemographic and HPV-knowledge data; recruitment via schools/universities.	Adolescent girls, young women and mothers. Mean age: -young women 19.1 -adolescents 15.4	Mean knowledge scores differed significantly: mothers 2.6, adolescents 3.6, young women 5.4. Young women had greater knowledge but emphasized the need for simpler messaging and guidance from healthcare professionals. Participants across groups expressed distrust towards the pharmaceutical industry and doubted the motivation of healthcare professionals in recommending vaccines.	Targeted HPV awareness campaigns should present information in a more concise and simple way, engage trusted healthcare providers to ensure effective communication, and provide more effective sexual health education to support evidence-based decisions about vaccination.
Restivo et al. (2018), Italy [27]	Cross-sectional study (telephone survey, Health Belief Model)	Telephone-administered structured questionnaire (23 items) conducted by trained healthcare professionals using vaccination registry data; targeted unvaccinated or partially vaccinated women	Females aged 18–21	84.4% were unvaccinated; 15.6% had received at least one dose. Vaccine refusal was associated with higher education; the lower level of education had better HPV awareness and higher rates of both HPV vaccination initiation and completion. The main reasons for refusing the HPV vaccine were lack of information (39.5%, n = 47), fear of adverse reactions to the vaccine (33.6%, n = 40), while 15.1% (n = 18) believed that the vaccine was not effective.	School-based educational interventions and enhanced physician counseling can improve vaccine acceptance and reduce refusal rates among young women. Unambiguous information about the efficacy, safety of vaccines, and the value of vaccination in preventing cervical cancer must be provided.
Vaidakis et al. (2017), Greece [22]	Cross-sectional epidemiological study (questionnaire)	Self-administered questionnaires distributed and collected in schools by health inspectors and research staff	Adolescents aged 17–18. Last year of secondary school students	42.8% were aware of HPV and 75.5% of cervical cancer, but over 60.6% were unaware of the association between the two or identified infection frequency and prevention. 21.1% were found without knowledge about methods of protection against HPV (condoms) and 37% for reducing the risk of cervical cancer. 40% knew about the HPV vaccine Female gender and religiosity were independent predictors of knowledge. Only 10.2% of girls had been vaccinated	Low awareness highlights the need for school-based sexual health education to strengthen HPV-related knowledge and prevention behaviors. Also, a paucity of systematic information regarding public opinion and health professionals has been identified.
McCutcheon et al. (2017), USA [28]	Cross-sectional study	Questionnaires before and after an HPV presentation	Male college athletes aged 18–23	68.4% had not received the HPV vaccine. 71% were sexually active, with 40% reporting multiple partners. 68.4% were aware of HPV. Of all the athletes surveyed, 97.4% reported having no history of STIs.	The study suggested that brief educational exposure was associated with increased knowledge and vaccination intent in male student populations.
Napolitano et al. (2016), Italy [25]	Cross-sectional study (knowledge and attitude survey)	Anonymous self-administered paper questionnaires completed by male students aged 14–24 in classroom settings. A variety of question types were included, such as multiple choice, 5-point and 10-point Likert scales, as well as open-ended items.	Males aged 14–24	54.9% were aware of HPV. The vast majority, 91.6% had awareness of the infection’s transmissibility during sexual intercourse. Only 15.2%, 6.7%, and 5.4% aware that HPV can cause cervical, anal, and oral cancers, respectively. 54.1% were aware of the vaccine. Knowledge was higher among sexually active males, those aware of the vaccine, and those informed by physicians. Only four students in this study had been vaccinated against HPV, and 58.2% expressed willingness to receive the vaccine.	Awareness campaigns targeting young men and enhancing physician communication could substantially increase male vaccination coverage.

Note: Studies including 14-year-old participants were retained when developmentally comparable to the predefined age range. Age-related consent and parental influence were considered during interpretation.

**Table 4:** Demographic Characteristics of Participants.

Study	Sample Size	Gender	Age (Mean, SD)	Occupation
Brunelli et al. (2025) [29]	463	Females (270; 58%) Males 193 (42%)	15.6 ± 1.8	Secondary school adolescents
Mekonnen et al. (2024) [26]	633	Females 100%	20.8 ± 0.75	Undergraduate health science students
Glenn et al. (2021) [18]	45	Women 33 (73%) Men 12 (27%)	21 ± 3	Undergraduate and graduate students
Karamanidou & Dimopoulos (2018) [19]	40	Women 100% -13 mothers -11 university students -16 adolescents.	Mean age: -mothers 47.8 -young women 19.1 -adolescents 15.4	Secondary school students and university students
Kasymova et al. (2019) [23]	256	Women 207 (80.9%) Men 49 (19.1%)	19.7 ± 1.7	University students
Kops et al. (2019) [24]	8581	Women 6366 (74.48%) Men 2182 (25.52%)	21.40	Young adults
McCutcheon et al. (2017) [28]	114	Men 100%	19.5 ± 1.29	Young athletes
Napolitano et al. (2016) [25]	956	Men 100%	19.6 ± 3.1	Secondary school and university students
Restivo et al. (2018) [27]	141	Women 100%	19	Vaccination program registry
Vaidakis et al. (2017) [22]	4547	Girls 2778 (61.6%) Boys 1729 (38.4%)	17.4 ± 0.6	Secondary school students

## Data Availability

All data generated or analysed during this study are included in this published article.
